# Teach-back: A systematic review of implementation and impacts

**DOI:** 10.1371/journal.pone.0231350

**Published:** 2020-04-14

**Authors:** Jason Talevski, Anna Wong Shee, Bodil Rasmussen, Georgie Kemp, Alison Beauchamp

**Affiliations:** 1 Department of Medicine–Western Health, The University of Melbourne, VIC, Australia; 2 Australian Institute for Musculoskeletal Science (AIMSS), The University of Melbourne and Western Health, VIC, Australia; 3 Ballarat Health Services, VIC, Australia; 4 Department of Rural Health, School of Medicine, Deakin University, VIC, Australia; 5 School of Nursing and Midwifery, Deakin University, VIC, Australia; 6 Centre for Quality and Patient Safety Research—Western Health Partnership, VIC, Australia; 7 Faculty of Health and Medical Sciences, University of Copenhagen, Copenhagen, Denmark; 8 School of Rural Health, Monash University, VIC, Australia; Universitat Witten/Herdecke, GERMANY

## Abstract

Patients often have difficulty comprehending or recalling information given to them by their healthcare providers. Use of ‘teach-back’ has been shown to improve patients’ knowledge and self-care abilities, however there is little guidance for healthcare services seeking to embed teach-back in their setting. This review aims to synthesize evidence about the translation of teach-back into practice including mode of delivery, use of implementation strategies and effectiveness. We searched Ovid Medline, CINAHL, Embase and The Cochrane Central Register of Controlled Trials for studies reporting the use of teach-back as an educational intervention, published up to July 2019. Two reviewers independently extracted study data and assessed methodologic quality. Implementation strategies were extracted into distinct categories established in the Implementation Expert Recommendations for Implementing Change (ERIC) project. Overall, 20 studies of moderate quality were included in this review (four rated high, nine rated moderate, seven rated weak). Studies were heterogeneous in terms of setting, population and outcomes. In most studies (n = 15), teach-back was delivered as part of a simple and structured educational approach. Implementation strategies were infrequently reported (n = 10 studies). The most used implementation strategies were training and education of stakeholders (n = 8), support for clinicians (n = 6) and use of audits and provider feedback (n = 4). Use of teach-back proved effective in 19 of the 20 studies, ranging from learning-related outcomes (e.g. knowledge recall and retention) to objective health-related outcomes (e.g. hospital re-admissions, quality of life). Teach-back was found to be effective across a wide range of settings, populations and outcome measures. While its mode of delivery is well-defined, strategies to support its translation into practice are not often described. Use of implementation strategies such as training and education of stakeholders and supporting clinicians during implementation may improve the uptake and sustainability of teach-back and achieve positive outcomes.

## Introduction

The healthcare system places a significant burden on patients to participate in their own care such as shared decision-making, providing informed consent or adhering to therapeutic regimens [1, 2]. Self-management of health is becoming increasingly complicated, leading to the need for strategies that support patients to not only understand complex health information, but also to apply this information in everyday life [3]. The ability to understand and use health information is a core component of health literacy, a concept which is consistently associated with health outcomes [4] and identified by the World Health Organization as key to achieving the Sustainable Development Goals [5].

There is a well-recognized communication gap in health care, with several studies identifying that healthcare providers may overestimate their own ability to communicate [6–8]. One survey-based study reported that 75% of surgeons believed they communicated well with their patients, but only 21% of their patients reported satisfactory communication [8]. Another qualitative study reported that 77% of doctors believed their patients were aware of their diagnosis, although only 57% of patients could correctly recall this [9]. These communication gaps can lead to adverse outcomes including compromised safety and increased economic burden [10, 11]. A frequently observed barrier to patient understanding is the continued use of medical terminology by doctors [12–14], with one systematic review reporting that patients want clearer explanations about their condition as they frequently misunderstand terms used in medical consultations [14]. Another major challenge in healthcare communication is patients’ ability to recall the information provided to them. Recall is considered an important mediator for treatment adherence and improved health outcomes [11, 15]. Studies have shown that less than half the information provided about medication and diet is accurately recalled by patients [15, 16], and can be even more challenging for people with low levels of education [3]. Interventions to improve communication at the patient-clinician interface are warranted; with one approach being the use of education and recall communication strategies such as ‘teach-back’ [17].

Teach-back involves asking patients to explain in their own words what a health provider has just told them. Any misunderstandings are then clarified by the health provider and understanding is checked again. This process continues until the patient can correctly recall the information that was given. Use of teach-back has been shown to improve knowledge, skills and self-care abilities in patients with chronic disease [18–25]. Teach-back is recommended as a health literacy-based communication approach in several policy documents and position statements, including the Australian Commission on Safety and Quality [17], the American Heart Association [26] and the American Diabetes Association [27]. Despite these recommendations and the simplicity of its use, teach-back is not consistently utilized [28–30]. This may, in part, be related to organizational or interpersonal barriers including lack of time, limited support by senior staff, or low self-efficacy to use teach-back [29, 31]. Furthermore, there is little guidance for healthcare services seeking to embed teach-back in their setting in a sustainable way. To promote the translation of teach-back into routine practice, it is important to identify strategies that may address any contextual and interpersonal barriers that support the uptake of this evidence-based intervention.

**Fig 1:**
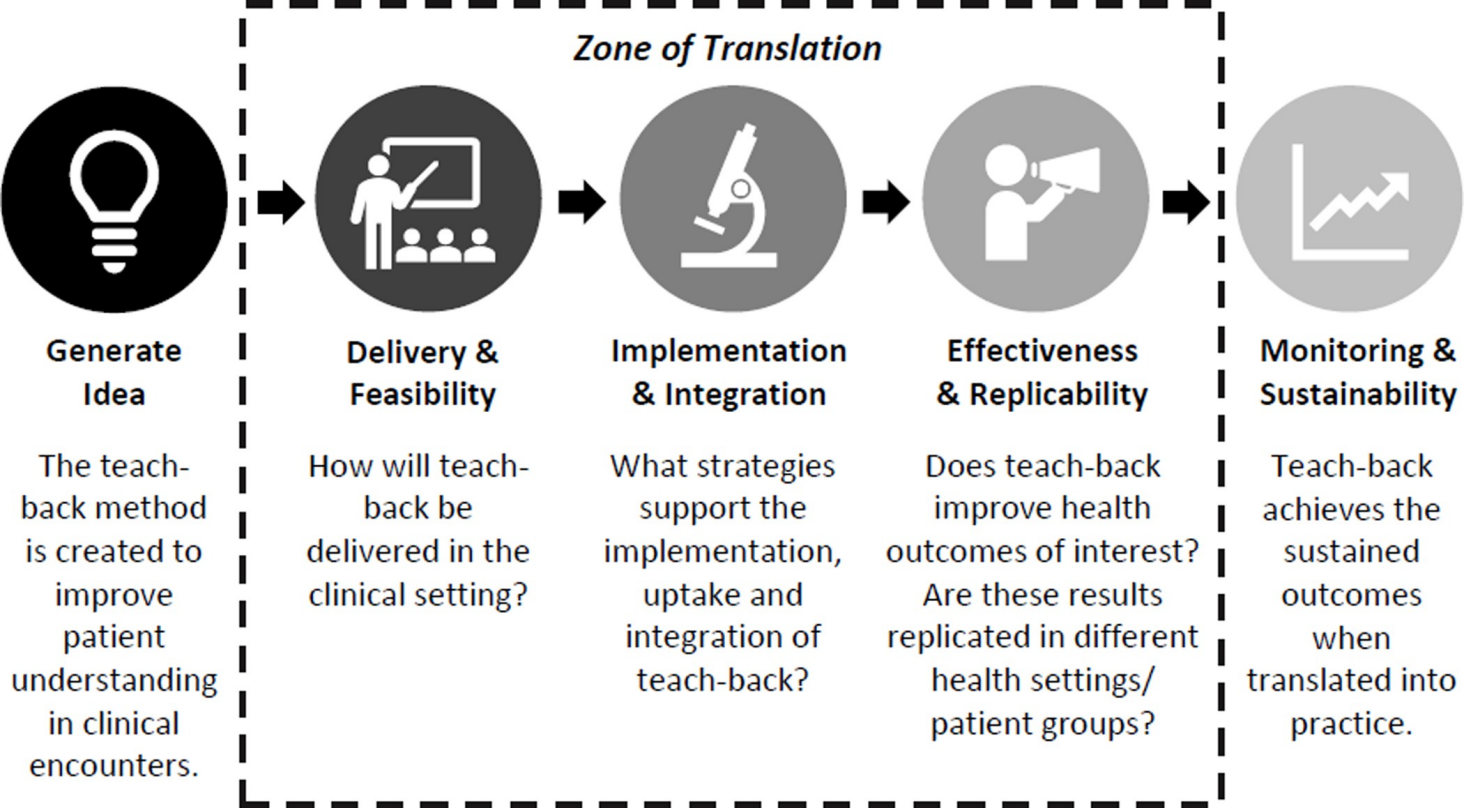
Translation of teach-back into practice; components evaluated in this review adapted from the Sax Institute’s Translational Research Framework [32].

This narrative review aims to synthesize the latest evidence about the translation of teach-back within healthcare settings including: 1) how teach-back is delivered in different settings; 2) what strategies are used to support the implementation and uptake of teach-back; and 3) the effectiveness of teach-back across different healthcare settings and populations. As shown in Fig 1, these components form the ‘zone of translation’ within the translational research process [32], thus providing a conceptual basis for our research questions.

## Material and methods

A systematic review was undertaken in accordance with the Preferred Reporting Items for Systematic reviews and Meta-Analyses (PRISMA) guidelines [33].

### Search strategy

An electronic search using Ovid Medline, CINAHL, Embase and The Cochrane Central Register of Controlled Trials was performed for published literature from inception to 11 July 2019. A sensitive search strategy was developed using the following search terms: “Teach-Back Communication” or “Teach-back” or “Show-me” or “Closing the loop” or “Closing the cycle” or “Ask-tell-ask” or “Repeat back” or “Verbal exchange” or “Patient-provider communication”. Reference lists from eligible studies, systematic reviews and grey literature were also reviewed for further relevant studies.

### Study selection

**Fig 2:**
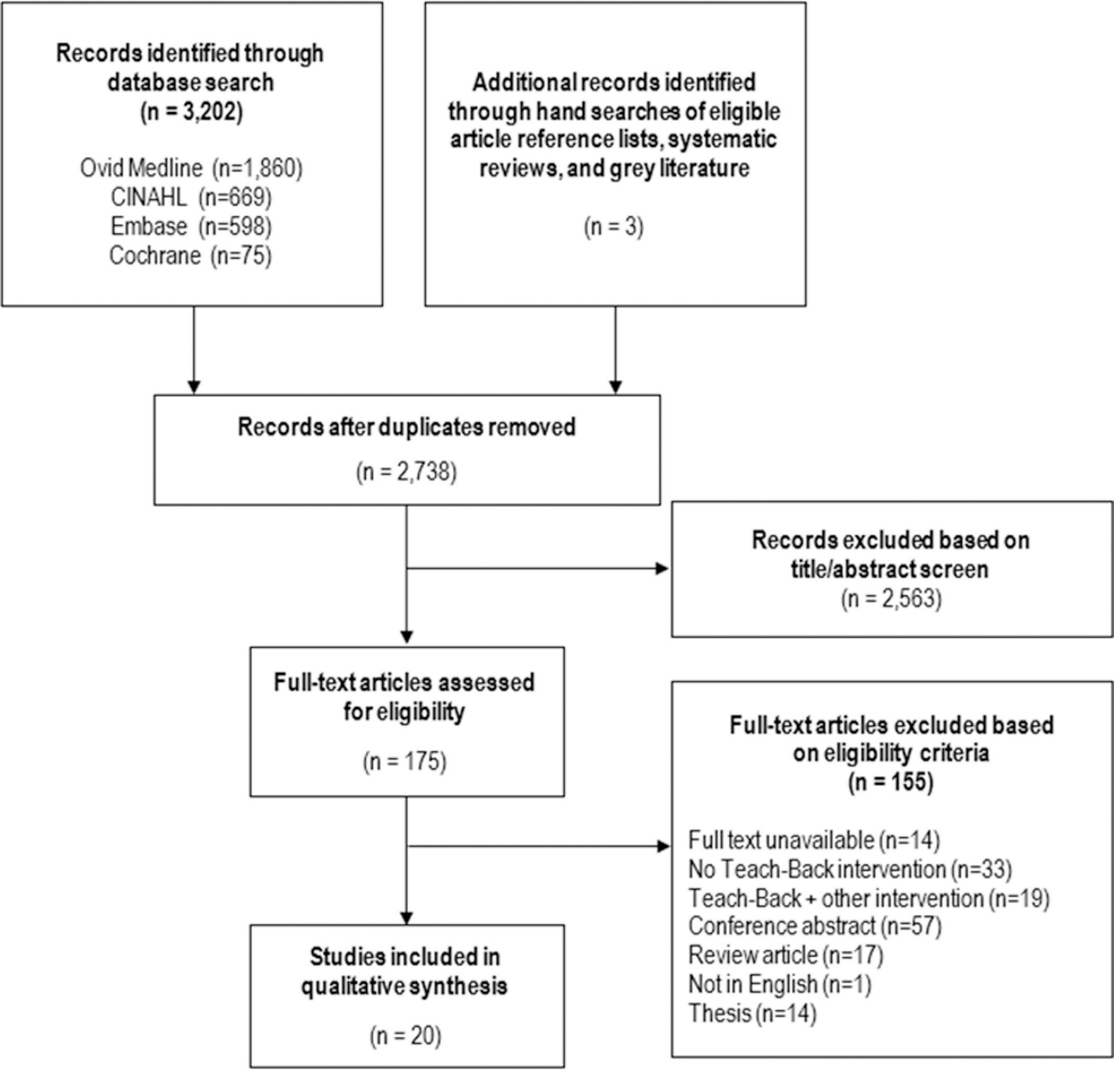
Study selection.

One author screened the titles and abstracts of potentially relevant studies against the eligibility criteria. Of potentially relevant studies identified from this initial screening, full length articles were attained and assessed independently by two authors. If there were discrepancies from the first two independent reviews, the authors discussed the conflicting results until consensus was reached. Reasons for exclusion at this stage were recorded and detailed in Fig 2.

### Eligibility criteria

#### Study design and participants

Studies were included if they were conducted as a randomized controlled trial (RCT), non-randomized trial, quasi-experimental study, case-control study, analytic cohort study or before and after study that implemented a teach-back intervention. This review included participants of all ages who were patients, clients or consumers with any health condition.

#### Teach-back intervention

Eligible studies included at least one group that participated in a teach-back intervention. A teach-back intervention was defined as ‘a structured education approach in which something is explained, the recipient’s understanding is checked by explaining back to the educator what they have just been told or demonstrating what they have been shown, any misunderstandings are then clarified, and understanding is checked again’. For the purposes of this review, structured education approaches were defined as those that were not complex in nature (i.e. not comprised of multiple, interacting components which were expected to lead to the primary outcome through several different pathways) [34]. Given that previous reviews have evaluated teach-back in conjunction with additional strategies (e.g. discharge care bundles, motivational interviewing) [18, 19], studies that delivered a teach-back intervention in combination with other comprehensive strategies were excluded from this review; unless the sole effect of teach-back could be extracted separately.

### Quality assessment

All studies were assessed for methodological quality by two independent reviewers using the Effective Public Health Practice Project (EPHPP) quality assessment tool for quantitative studies [35]. This tool was developed for use in RCTs, controlled clinical trials, case-control and observational study designs and includes eight domains of quality assessment: selection bias (were participants representative of the target population); study design; confounders (controlled for in the analysis); blinding (outcome assessors/participants); data collection methods (use of valid tools); withdrawals and dropouts; intervention integrity (consistency of the intervention); and analysis (use of appropriate statistical methods). The final two domains are not included in the overall methodological quality score. The EPHPP tool leads to an overall methodological rating score of strong, moderate or weak, and has been evaluated for content and initial construct validity and inter-rater reliability [35]. If consensus could not be reached by the two independent reviewers, a third reviewer was called upon to complete an independent quality assessment.

### Data synthesis

Data from included studies were independently extracted by two authors. The following information was extracted from each study: lead author, publication year, country of study, study design, participant characteristics (% female, mean age, health condition), intervention description, outcome data (outcome measures and effect size), mode of delivery and implementation strategies. Implementation strategies were extracted into distinct categories established in the Implementation Expert Recommendations for Implementing Change (ERIC) project [36, 37]. In the ERIC project, a panel of experts in the field of implementation science and clinical practice compiled a list of 73 implementation strategies and grouped them into 9 categories, with the intention of guiding implementation research and clinical practice (S1 Table). The extracted data were evaluated against the ERIC framework to determine how teach-back has been previously implemented within healthcare settings. Data synthesis was primarily done by the first author and checked for consistency by the corresponding author.

## Results

### Literature search

The electronic search identified 2,738 studies for screening of eligibility after duplicate studies were removed. Of these, 2,563 studies were excluded based on title and abstract and full text was obtained for the remaining 175 studies. Based on the authors’ decisions, 20 studies met the eligibility criteria and were included in this review (Fig 2).

### Study characteristics

Of the 20 studies, there were nine RCTs, two controlled clinical trials, four pre-post studies, one before and after study, three prospective cohort studies and one cross-sectional study. Most studies were from the USA (n = 10) or Iran (n = 7); with one study each from Australia, China and India. Studies were conducted across hospitals (n = 8), emergency departments (EDs; n = 3), outpatient clinics (n = 4), primary care practices (n = 2), community health centers (n = 1) and nursing homes (n = 1). There was a broad range of participant characteristics across studies. One study included children aged 6–13 years old; two studies focused on older adults aged ≥60 years; one study included young adults aged 20–30 years; two studies included people aged 30–55 years; and 15 studies included all adults aged ≥18 years. Studies included people with chronic conditions such as heart disease (n = 2), chronic obstructive pulmonary disease (n = 2), Type II Diabetes (n = 4), breast cancer (n = 1) and asthma (n = 2); post-surgical inpatients (n = 2); and people discharged from the ED (n = 2). Eleven studies included a higher percentage of females. Patient characteristics are summarized in Table 1.

**Table 1:** Study and participant characteristics.

Author (year)	Setting	Study design	Participants				Primary Outcome(s)	Key Findings	Study Quality
			N (I/C)	Age (years), mean (±SD)	% Female	Population/ Condition			
Ahmadidarrehsima et al. (2016) [38]	Hospital, Iran	Controlled Clinical Trial	50 (25/25)	I: 56(46–60)^†^ C: 40 (40–54)^†^	100%	Breast Cancer	Happiness	↑ Oxford Happiness Inventory score in the TB group compared to the control group immediately post intervention (62.9 vs 29.8; p<0.001).	Strong
Ahrens & Wigres (2013) [3]	Hospital, USA	Before & After	121 (60/61)	NR	NR	Neurological Patients	Patient Satisfaction	↑ Patient satisfaction from 29.7% to 77.3% post-TB implementation.	Moderate
Badaczewski et al. (2017) [40]	Paediatric Care Centre, USA	Cross-sectional	44	9 (6–13)^†^	34%	Asthma	Patient-centered Interactions	↑ Patient-centered communication (OR = 4.97; 95% CI: 4.47–5.53) and engagement of parents during pediatric clinical encounters following TB.	Weak
Bahri et al. (2018) [41]	Community Health Centre, Iran	RCT	66 (32/34)	I: 53.5 (1.5) C: 53.3 (1.6)	100%	Post-menopausal Women	Self-care Management	↑ Knowledge about self-care and self-care activities in the TB group compared to the control group 1 month after the intervention (p<0.001).	Moderate
George et al. (2018) [42]	Hospital, India	Before & After	98	18–70^‡^	38%	Type II diabetes	Medication Adherence	Patients in the low and medium medication adherence groups showed an improvement in medication adherence 2 months post-TB (p<0.05). Patients who were categorized in the high adherence group did not show any change after TB (that is they continued to be highly adherent).	Weak
Ghiasvand et al. (2017) [43]	Hospital, Iran	RCT	80 (40/40)	I: 24.5 (4.5) C: 24.5 (4.5)	100%	Women with Post-partum Depression	Quality of Life	↑ Post-partum quality of life in the TB group compared to control group at 8-week follow-up (124.7 vs 115.0; p<0.001).	Moderate
Griffey et al. (2015) [44]	Emergency Department, USA	RCT	408 (212/196)	I: 36.0 (13.2) C: 34.7 (12.8)	60%	Low Health Literacy	Comprehension & Satisfaction	↑ Comprehension of post-ED medications (p<0.02), self-care (p<0.03) and follow-up instructions (p<0.001) in TB patients compared to standard care post-intervention. ↔ Comprehension or patient satisfaction between groups.	Strong
Haney & Shepherd (2014) [45]	Hospital, USA	Prospective Cohort	23	NR	NR	Heart Failure (high-risk)	Hospital Re-admissions	↓ 30-day re-admission rate from 18% to 13% over 6-months.	Weak
Kandula et al. (2011) [46]	Outpatient Clinic, USA	Two-Group, Pre-Post Study	171 (58/113)	I: 52.8 (9.7) C: 56.2 (9.9)	75%	Type II Diabetes	Knowledge Retention	↑ Immediate recall of information in the TB + Education vs Education Only group. ↔ Compared to education only, TB + Education did not improve knowledge retention at 2-week follow-up.	Weak
Kiser et al. (2012) [47]	Primary Care Centre, USA	RCT	99 (67/32)	I: 63 (43–84)^†^ C: 63 (44–83)^†^	65%	COPD	Inhaler Technique	↑ Inhaler technique score in the TB group compared to usual care group (mean change: 1.6 vs -0.5; p<0.001).	Moderate
Liu et al. (2018) [48]	Nursing Home, China	RCT	260 (126/134)	I: 79.2 (8.8) C: 79.1 (9.2)	49%	Older People	Health Literacy	↑ Health literacy score in the TB group compared to the control group immediately post-intervention (110.1 vs 74.9; p<0.001).	Strong
Mahmoudir-ad et al. (2015) [49]	Outpatient Clinic, Iran	Controlled Clinical Trial	70 (35/35)	I: 51.8 (4.2) C: 50.0 (5.6)	75%	Type II Diabetes	Self-care Management	↑ Foot self-care scores in the TB group compared to the control group at 3-month follow-up (29.3 vs 19.2; p<0.001).	Moderate
Moadab et al. (2015) [50]	Hospital, Iran	RCT	60 (30/30)	I: 25.2 (2.9) C: 25.5 (2.7)	100%	Post-Caesarean Surgery	Anxiety	↓ Anxiety level scores in patients post-TB compared to control patients awaiting caesarean surgery (50.8 vs 59.4; p<0.001).	Moderate
Mollazadeh & Maslakpak (2018) [51]	Outpatient Clinic, Iran	RCT	84 (42/42)	I: 38 (12.4) C: 41.3 (11.7)	33%	Kidney Transplant Recipients	Self-care Management	↑ Self-management scores in the TB group compared to the control group at 2-month follow-up (82.5 vs 74.4; p<0.001).	Strong
Morony et al. (2018) [52]	Telephone Call Centre, Australia	RCT	637 (261/376)	I: 31.1 (6.4) C: 31.5 (6.6)	87%	Telephone Health Service Users	Self-care Management	↑ Confidence to act (OR = 2.44; p = 0.06) and knowledge of healthcare services (OR = 2.68; p = 0.06) in TB callers compared to control group callers.	Moderate
Negarandeh et al. (2013) [53]	Outpatient Clinic, Iran	RCT	83 (43/40)	I: 50.3 (8.5) C: 49.1 (8.8)	46%	Type II Diabetes	Knowledge Retention & Medication/ Diet Adherence	↑ Mean scores of knowledge, and adherence to medication and diet in the TB group compared to the control group at 6-week follow-up (p< 0.001).	Moderate
Peter et al. (2015) [54]	Hospital, USA	Two-Group, Pre-Post Study	469 (180/289)	NR	NR	Heart Failure (high-risk)	Comprehension & Hospital Re-admissions	↑ Patient understanding of their disease. ↓ 12% in re-admission rates for heart failure patients 1 years post-TB implementation.	Weak
Press et al. (2011) [55]	Hospital, USA	Prospective Cohort	42	51.7 (17.4)	73%	COPD & Asthma	Inhaler Technique	After one round of TB, 86% of participants achieved correct inhaler use. After a second-round of TB, all participants achieved correct use.	Moderate
Slater et al. (2017) [56]	Emergency Department, USA	Two-Group, Pre-Post Study	209 (105/104)	I: 38.0 (14.0) C: 41.0 (18.0)	68%	Emergency Department Patients	Knowledge Retention	↑ Retention of discharge instructions (diagnosis, medications, follow-up instructions) in the TB group compared to the control group (recall rate 82.1% vs 70.0%; p<0.05).	Weak
Waszak et al. (2018) [57]	Emergency Department, USA	Prospective Cohort	52	NR	NR	Emergency Department Patients	Knowledge Retention	100% of patients clearly understood how to take opioids, and 80.8% learned something new about how to take, store, or dispose of their medications safely.	Weak

I = Intervention Group; C = Control Group; SD = Standard Deviation; COPD = Chronic obstructive pulmonary disease; TB = Teach-Back; NR = Not reported.

^†^Median (Interquartile range).

^‡^Range.

### Methodological quality

The methodological quality of included studies varied (Tables 1 and S2). Four studies were rated as high quality, nine as moderate quality and seven as weak. Common methodologic limitations identified across studies included omission of reporting if outcome assessors were blinded to intervention/exposure study of participants, participants being blinded to the research questions and whether individuals selected to participate in the study were likely to be representative of the target population.

### Delivery of teach-back

Teach-back interventions were delivered by either healthcare personnel, including nurses (n = 9), primary care providers (n = 2) and pharmacists (n = 1); or by research staff (n = 8). Fifteen studies [38, 39, 41, 43, 45–51, 53–55, 57] used teach-back as part of a structured educational approach. This teach-back enhanced education ranged from brief sessions to more complex training. Nine studies [38, 41, 43, 46, 48, 49, 51, 53, 54] comprised more than one education session over the study period (range 2–11); brochures or written information were given in five studies [39, 42, 47, 53, 57] to aid the teach-back process; and demonstration of a technique was used in two studies [47, 55]. There were four studies in which teach-back was delivered as a less structured approach; two of these focused on training clinicians to use teach-back as part of their routine care [31, 40], and two studies [44, 56] trained nursing staff to use teach-back when providing discharge instructions in the ED (Table 2).

**Table 2:** Implementation characteristics of teach-back.

Author (year)	Person(s) Delivering Teach-Back	Mode of Delivery	Implementation Strategies (36, 37)								
			Evaluative & Iterative Strategies	Provide Interactive Assistance	Adapt & Tailor to Context	Develop Stakeholder Inter-relationships	Train & Educate Stakeholders	Support Clinicians	Engage Consumers	Utilize Financial Strategies	Change Infra-structure
Ahmadidarrehsima et al. (2016) [38]	Researcher	8–11 breast cancer self-management education sessions with TB lasting 1.5 to 2 hours.	-	-	-	-	-	-	-	-	-
Ahrens & Wigres (2013) [39]	Nurse	Verbal and written medication side-effect education with TB implemented during hourly rounding (the ‘‘Always Ask” program).	✓			✓	✓	✓			
Badaczewski et al. (2017) [40]	Primary Care Provider	Primary care clinicians were provided with 3 one-hour interactive training sessions with TB on health literacy. TB occurrence during clinical encounters was measured using a Teach-back Loop Score.					✓	✓			
Bahri et al. (2018) [41]	Researcher	Four 45-minute education sessions with TB on menopausal self-care using a video projector.	-	-	-	-	-	-	-	-	-
George et al. (2018) [42]	Pharmacist	Medium adherent patients were provided with a medication information leaflet and patient counseling with TB; Low adherent patients were provided with audio-visual aids, a medication information leaflet and patient counseling with TB.	NR	NR	NR	NR	NR	NR	NR	NR	NR
Ghiasvand et al. (2017) [43]	Researcher	Two 1-hour education sessions with TB on post-partum self-care.	-	-	-	-	-	-	-	-	-
Griffey et al. (2015) [44]	Nurse + Researcher	Standard discharge instructions with TB.		✓			✓				✓
Haney & Shepherd (2014) [45]	Nurse	One 60-minute self-management education session with TB.	NR	NR	NR	NR	NR	NR	NR	NR	NR
Kandula et al. (2011) [46]	Researcher	Two video education modules on Diabetes with TB.	-	-	-	-	-	-	-	-	-
Kiser et al. (2012) [47]	Researcher	One education session of correct inhaler use including visual demonstration with TB + a take-home information sheet.	-	-	-	-	-	-	-	-	-
Liu et al. (2018) [48]	Graduate Students	Two health literacy education sessions with TB delivered over 6-months.					✓		✓	✓	
Mahmoudirad et al. (2015) [49]	Researcher	Up to three 45-minute education sessions on foot self-care with TB.	-	-	-	-	-	-	-	-	-
Moadab et al. (2015) [50]	Primary Care Provider	One 30-45-minute tailored education session on caesarian surgery with TB pre-op.	NR	NR	NR	NR	NR	NR	NR	NR	NR
Mollazadeh & Maslakpak (2018) [51]	Nurse	Five 60-minute tailored self-care education sessions with TB over 3 months.	✓								
Morony et al. (2018) [52]	Nurse	Telephone callers (nurses) were trained in using TB with callers when providing comprehensive information and advice.	✓				✓	✓			
Negarandeh et al. (2013) [53]	Nurse	Three 20-minute Diabetes education sessions with TB + a take-home information sheet.									
Peter et al. (2015) [54]	Nurse	TB-enhanced, structured education on heart failure self-care over 3-days.	✓	✓		✓	✓	✓			✓
Press et al. (2011) [55]	Researcher	One education session of correct inhaler use including visual demonstration with TB.	-	-	-	-	-	-	-	-	-
Slater et al. (2017) [56]	Nurse	Standard discharge instructions with TB.					✓	✓			
Waszak et al. (2018) [57]	Nurse	One dual-modal (verbal and written) education session on opioid use with TB.					✓	✓			✓

- = Not Relevant (researcher-led implementation); NR = Not Reported; TB = Teach-back.

### Implementation strategies

Implementation strategies were infrequently reported within studies. Of the 20 included studies, only 10 [39, 40, 44, 48, 51–54, 56, 57] reported sufficient information regarding implementation of teach-back. Of the remaining 10 studies, three [42, 45, 50] did not report any details related to implementation and seven [38, 41, 43, 46, 47, 49, 55] delivered teach-back via research staff, meaning that implementation strategies were not relevant in clinical practice, but for research purposes only (Table 2).

#### Train and educate stakeholders

Providing training and education to staff who were employing teach-back was the most used implementation strategy (n = 8 studies) [39, 40, 44, 48, 52, 54, 56, 57]. Education and training programs differed but generally focused on four main concepts: 1) identifying the needs of the patient; 2) establishing the preferred learning style of the patient; 3) choosing the appropriate resources; and 4) demonstration of how to use teach-back during a patient interaction. Seven studies [39, 44, 48, 52, 54, 56, 57] provided a one-time education/training program in teach-back, and one study [40] provided three 1-hour interactive training sessions in health literacy and use of teach-back. One study [54] utilized an online education module, five studies [44, 48, 52, 56, 57] implemented a group education session, three studies [40, 44,56] provided interactive role-playing scenarios and one study provided written education materials [39].

#### Use of evaluative and iterative strategies

Four studies [39, 51, 52, 54] reported the use of evaluative and iterative strategies. Two of the studies [52, 54] developed a quality monitoring system to ensure teach-back was being undertaken appropriately, with one of these [54] also providing continued auditing of the teach-back standard. One study [39] implemented a weekly email update to inform nurses delivering teach-back on their progress and improvements, and one study [51] developed a needs assessment checklist to inform a targeted education approach for each patient.

#### Provide interactive assistance

Providing interactive or technical assistance were reported in two studies [44, 54]. This included techniques such as documentation and tracking of the use of teach-back encounters via patient electronic medical records (EMRs).

#### Adapt and tailor to context

No studies reported an interpersonal focus by tailoring or adapting the teach-back intervention to the specific patient population.

#### Develop stakeholder interrelationships

Developing stakeholder relationships, an important process for sustainability, was reported in only two studies [39, 54]. One of these studies [54] designated teach-back champions on each ward to guide and motivate nurses in the use of teach-back. These champions were provided with an additional 2-hour ‘‘train-the-trainer” workshop. The other study [39] organized implementation team meetings between nurses (administers of teach-back) and a newly convened patient perception team (made up of managers, bedside nurses, and individuals who had been treated at the hospital) to achieve nursing feedback and support for the intervention.

#### Support for clinicians

Ongoing support for clinicians implementing teach-back was reported in six studies [39, 40, 52, 54, 56, 57] and mainly focused on developing clinical reminders for teach-back use. This included use of prompts such as posters and flyers on the wards [39, 40, 56, 57], reminder cards [56], notes on white boards in patients’ rooms [54] and electronic prompting processes such as reminder emails or videos [39, 52, 57].

#### Engage consumers

Only one study [48] engaged patients in the development of an implementation plan for the delivery of teach-back. This study solicited feedback from recipients through interviews on the teach-back process to inform the delivery of the intervention moving forward.

#### Changes in infrastructure

Three studies [44, 54, 57] employed a change in infrastructure, primarily through the EMR system. This included adding teach-back prompts to the EMR [40, 54] and making patient education materials and documentation of teach-back available within the EMR [57].

#### Utilize financial strategies

Utilizing financial strategies was one of the least utilized implementation strategies, applied in only one study [48]. This study organized a prize-winning knowledge contest among patients each month as an incentive to reinforce the educational effect of teach-back and stimulate interest in patients to participate.

### Outcome measures and effectiveness of teach-back

Although there was variability among studies in relation to study populations, settings and outcomes, 19 studies (95%) reported positive findings for primary outcome measures. The outcomes measured fell into three distinct categories: 1) knowledge, skills and attitudes (disease knowledge, comprehension and retention, patient satisfaction); 2) behavior change (self-care practices, medication adherence); and 3) objective health-related outcomes (hospital re-admissions, quality of life). Three studies [44, 48, 52] measured health literacy, however only one study measured health literacy change as an outcome [48]. In this RCT of older people (aged ≥60 years), health literacy scores–measured by the Chinese Citizen Health Literacy Questionnaire–significantly increased in the teach-back group compared to the control group (110.1 vs 74.9; p = 0.001) [48]. The three studies that involved patients discharged from ED [44, 56, 57] reported increased knowledge of post-discharge procedures after teach-back compared to standard discharge instructions. Most studies were conducted among participants with chronic conditions (n = 12). Studies that included participants with Type II diabetes reported significant improvements in medication adherence [42, 53], diet changes [53] and foot self-care [49] following teach-back compared to usual care control groups. The two studies in heart failure patients measured hospital re-admissions, with both studies reporting a minor reduction in re-admission rates [45, 54]. Demonstration of proper inhaler technique using teach-back in people with chronic obstructive pulmonary disease lead to significant improvements in inhaler technique in two studies [47, 55]. One study in children with asthma found that teach-back was associated with increased patient-centered communication (OR = 4.97; 95% CI: 4.47–5.53) and increased engagement of parents during pediatric clinical encounters [40]. Other outcomes showing improvements following teach-back included happiness in breast cancer patients [38], quality of life in post-partum women [43], anxiety in women awaiting caesarean surgery [50] and patient satisfaction in participants using a maternal and child health call center [52]. Most outcomes were measured immediately post-intervention (n = 11); studies with follow-up ranged from 2 weeks to 1 year. Outcome measures and key findings are summarized in Table 1.

## Discussion

To our knowledge, this is the first systematic review appraising the translation of teach-back into clinical practice. We found that implementation of teach-back is not well described in the literature. The most frequently utilized implementation strategies were training and education of stakeholders (e.g. educational materials, training modules) and reminding clinicians to implement the intervention (e.g. clinical reminders/prompts). Findings from this review can inform healthcare services and providers about key strategies to optimize the routine uptake and sustainability of this effective health literacy-based communication technique.

### Delivery and feasibility

Teach-back was most commonly delivered as part of a structured, but simple educational approach, with this ‘teach-back enhanced education’ being reported as effective across a wide range of settings, populations and outcome measures. Settings included hospitals, outpatient clinics, the ED, and community health centers. Many health interventions are designed for a specific setting and are generally not implemented in a different setting to that which it was intended for [58]. Findings from this review reflect the broad application of teach-back enhanced education across multiple settings, including the ED. Delivering health interventions or programs in the ED is challenging given the high-pace and perplexing nature of this setting. Previous studies have shown that ED clinicians rarely confirm comprehension of instructions with their patients and that patient comprehension of ED discharge instructions is poor [59, 60]. Three studies in this review were undertaken in the ED setting [44, 56, 57] and reported increased knowledge of post-discharge procedures, higher levels of diagnosis knowledge and improved recall of follow-up instructions compared to standard discharge care. These results highlight the success of teach-back in reinforcing ED discharge instructions and should be considered by ED clinicians as a key component when providing patients with information.

### Implementation and integration

The most used implementation strategy in the included studies was training and educating the healthcare providers who were delivering teach-back. Education sessions were often structured and focused on the importance of tailoring teach-back to patient needs, reflecting best-practice communication techniques [61]. However, while education is essential to introduce a new intervention it is well-established that training alone is not sufficient to effect ongoing change and uptake into standard clinical practice [62]. Successful implementation requires a multifaceted approach that is guided by an implementation plan or framework, and incorporates an identified need for improvement, collaboration between stakeholders and health services, flexibility in responding to feedback, using data to drive practice change, and a culture receptive to change [63]. Among the studies in this review, only one reported using almost all implementation strategies in the ERIC framework [54]. The authors took a staged approach to implementation, initially establishing a multidisciplinary working group. This group developed a structured teach-back protocol; clinicians were trained in identification of the key learner for each patient and educated in use of teach-back through an online learning module; teach-back prompts and feedback were provided within the patient EMR; and teach-back champions were trained and assigned to individual wards. This detailed and systematic implementation plan resulted in significant improvements in patients’ understanding of their disease, improved compliance among nurses regarding the use of teach-back in educating patients, and a sustained drop in readmission rates for patients with heart failure one-year post-implementation.

The lack of involvement from consumers in the implementation of teach-back was surprising, given the consumer-focus of teach-back and the current global interest in the involvement of consumers in the design and implementation of healthcare interventions. Three studies [51, 53, 54] mentioned assessing the patients’ understanding before providing teach-back in order to tailor the intervention to fit the individual’s learning needs, however this is part of standard teach-back practice.

In terms of process evaluation, no studies in this review assessed implementation fidelity. This concept refers to the extent to which an intervention has been implemented in practice as it was intended to [64]. Implementation fidelity has been frequently recommended as an essential component of undertaking intervention trials [65], yet was not examined in any of the studies included in this review. Therefore, a key message from our review is that there is a need to improve reporting of implementation fidelity within intervention trials assessing the teach-back method. This would allow researchers and clinicians to identify the association between successful implementation and improved outcomes and promote the integration of teach-back into routine practice.

### Effectiveness and replicability

The overwhelming effectiveness of teach-back reported in 95% of studies, and across such a broad range of patient groups and outcomes, supports the use of teach-back enhanced education in clinical practice. Additionally, the studies were of moderate quality which limits the degree of caution for interpreting this conclusion. Systematic reviews of studies examining educational interventions (without teach-back) have shown inconclusive or even negative findings [66, 67]. Most studies in this review delivered teach-back as part of a simple educational program and compared outcomes against participants receiving ‘general education’. This demonstrates that teach-back is a valuable addition to patient education in health care settings. However, most learning-related outcomes were measured immediately post-intervention; therefore, research demonstrating that teach-back has long-term effects on patient knowledge and recall is warranted. Further, outcomes such as patient knowledge, recall of information and medication adherence are feasible outcome measures within healthcare settings. Providing feedback to clinicians through demonstration of positive outcomes may be one useful strategy to build support for continued use of teach-back.

Finally, comprehending medical diagnoses and treatments requires a level of intermediate or proficient health literacy. It is well established that patients with low health literacy have less ability to understand and recall health information [68, 69], although few studies examined the effectiveness of teach-back to improve comprehension and recall across patients with differing literacy levels. Three studies in this review [44, 48, 52] measured health literacy in participants, although only *Liu et al*. measured health literacy change as an outcome. In this RCT of older people (aged ≥60 years), health literacy scores significantly increased in the teach-back group compared to the control group [48]. A second RCT reported that teach-back significantly improved comprehension of post-ED care (i.e. medications, self-care, and follow-up instructions) among patients with limited health literacy, compared to standard ED discharge instructions [44]. Similarly, *Morony et al*. reported improved knowledge of healthcare services among people with inadequate health literacy following a nurse-delivered teach-back teleconsultation [52]. Health providers are the most trusted source of health information for people [70], and therefore have a responsibility to deliver information to their patients that is clear, understandable and practical [71]. This is especially true for those with limited literacy skills, who are more likely to solely rely on their clinician for health information [72]. Teach-back may be a feasible, practical and cost-effective intervention to address this health literacy-based communication gap in health care.

### Strengths and limitations

The strengths of this review include our rigorous methodology and comprehensive search strategy. We have confidence that we identified all published studies that met our inclusion criteria as we used various synonyms of “teach-back” in our search strategy. Furthermore, we excluded studies that delivered a teach-back intervention in combination with other comprehensive strategies so we could examine the sole effect of teach-back. This differed from all previous reviews on the teach-back method [18, 19]. Limitations of our review should also be considered. Searches were limited to published studies, subjecting this review to the possibility of publication bias. Only half of the included studies provided a detailed description of implementation–three studies provided no information on implementation and teach-back was delivered by research staff in seven studies (implementation strategies not relevant in clinical practice)–which limited the main aim of this review. Additionally, given the heterogeneity of outcome measures, it was not possible to conduct a meta-analysis. It would be useful to understand the link between implementation and health outcomes; however, this was not possible due to the lack of detail regarding implementation and heterogeneity of implementation strategies in the included studies.

## Conclusions

Teach-back is effective across a wide range of settings, populations and outcome measures, although implementation techniques are not well described. Use of recognized implementation strategies such as training and education of stakeholders (e.g. educational materials, training modules) and supporting clinicians to apply the intervention (e.g. clinical reminders/prompts) may support the uptake and sustainability of teach-back. In clinical practice, teach-back provides a low-cost and effective technique that can be used to enhance structured, simple education to achieve positive outcomes in communication at the patient-clinician interface. Further research examining the long-term benefits and barriers to translation of teach-back is recommended.

## Supporting information

S1 Table Implementation categories from the Expert Recommendations for Implementing Change (ERIC) project (DOCX)

S2 Table Quality assessment results using the Effective Public Health Practice Project (EPHPP) tool (DOCX)

S1 Checklist PRISMA guidelines (DOC)
