# Supplementary material for: Teach-back: A systematic review of implementation and impacts
Source: PLoS One. 2020 Apr 14;15(4):e0231350. doi: 10.1371/journal.pone.0231350 (PMC7156054; doi:10.1371/journal.pone.0231350)
Supplement: S1 Table — (DOCX) [file pone.0231350.s001.docx]

**Implementation categories from the Expert Recommendations for Implementing Change (ERIC) project**

| **Implementation category** | **Implementation strategies** |
| --- | --- |
| 1) Use evaluative and iterative strategies | - Assess for readiness and identify barriers and facilitators - Audit and provide feedback - Develop and implement tools for quality monitoring - Develop and organize quality monitoring systems - Conduct local needs assessment - Stage implementation scale up - Obtain and use patients/consumers and family feedback - Conduct cyclical small tests of change |
| 2) Provide interactive assistance | - Provide/centralize local technical assistance - Provide clinical supervision |
| 3) Adapt and tailor to context | - Tailor strategies and promote adaptability - Use data experts and data warehousing techniques |
| 4) Develop stakeholder interrelationships | - Identify champions and implementation advisors - Organize clinician implementation team meetings - Recruit, designate, and train for leadership - Inform local opinion leaders and build a coalition - Conduct local consensus discussions - Capture and share local knowledge - Use advisory boards and workgroups - Develop academic partnerships (network weaving) |
| 5) Train and educate stakeholders | - Provide ongoing consultation and training - Develop and distribute educational materials - Use train-the-trainer strategies - Conduct educational meetings and outreach visits; - Shadow other experts |
| 6) Support clinicians | - Facilitate relay of clinical data to providers - Prompts and reminders for clinicians - Develop resource sharing agreements - Revise professional roles and create new clinical teams |
| 7) Engage consumers | - Involve patients/consumers and family members - Prepare patients/consumers to be active participants |
| 8) Utilize financial strategies | - Fund and contract for the clinical innovation - Place innovation on fee for service lists/formularies - Alter incentive/allowance structures - Alter patient/consumer fees - Use other payment schemes |
| 9) Change infrastructure | - Change record systems, physical structure and equipment - Create or change credentialing and/or licensure standards - Change service sites - Change accreditation or membership requirements - Start a dissemination organization |
