# Supplementary material for: Teach-back: A systematic review of implementation and impacts
Source: PLoS One. 2020 Apr 14;15(4):e0231350. doi: 10.1371/journal.pone.0231350 (PMC7156054; doi:10.1371/journal.pone.0231350)
Supplement: S2 Table — (DOCX) [file pone.0231350.s002.docx]

**Quality assessment results using the** **Effective Public Health Practice Project (EPHPP) tool**

| **Author**  **(year)** | **Domains** | | | | | | **Overall**  **Quality**  **Score** |
| --- | --- | --- | --- | --- | --- | --- | --- |
|  | **Selection**  **Bias** | **Study**  **Design** | **Confounders** | **Blinding** | **Data Collection** | **Withdrawals & Dropouts** |  |
| Ahmadidarrehsima et al. (2016) | Moderate | Strong | Strong | Moderate | Strong | Not Applicable | **Strong** |
| Ahrens & Wigres (2013) | Moderate | Moderate | Not Applicable | Weak | Strong | Moderate | **Moderate** |
| Badaczewski et al. (2017 | Weak | Weak | Weak | Moderate | Strong | Not Applicable | **Weak** |
| Bahri et al. (2018) | Moderate | Strong | Strong | Weak | Strong | Strong | **Moderate** |
| George et al. (2018 | Moderate | Moderate | Weak | Weak | Strong | Strong | **Weak** |
| Ghiasvand et al. (2017) | Moderate | Strong | Strong | Moderate | Strong | Weak | **Moderate** |
| Griffey et al. (2015) | Moderate | Strong | Strong | Moderate | Strong | Moderate | **Strong** |
| Haney & Shepherd (2014) | Moderate | Moderate | Not Applicable | Weak | Weak | Weak | **Weak** |
| Kandula et al. (2011) | Weak | Moderate | Strong | Weak | Strong | Moderate | **Weak** |
| Kiser et al. (2012) | Weak | Strong | Strong | Moderate | Strong | Moderate | **Moderate** |
| Liu et al. (2018 | Moderate | Strong | Strong | Moderate | Strong | Strong | **Strong** |
| Mahmoudirad et al. (2015) | Moderate | Strong | Strong | Weak | Strong | Moderate | **Moderate** |
| Moadab et al. (2015) | Strong | Strong | Weak | Strong | Strong | Strong | **Moderate** |
| Mollazadeh & Maslakpak (2018) | Moderate | Strong | Strong | Strong | Strong | Strong | **Strong** |
| Morony et al. (2018) | Moderate | Strong | Strong | Strong | Strong | Weak | **Moderate** |
| Negarandeh et al. (2013) | Moderate | Strong | Strong | Weak | Strong | Strong | **Moderate** |
| Peter et al. (2015) | Weak | Moderate | Weak | Weak | Strong | Weak | **Weak** |
| Press et al. (2011) | Moderate | Moderate | Not Applicable | Weak | Moderate | Not Applicable | **Moderate** |
| Slater et al. (2017) | Weak | Moderate | Strong | Weak | Weak | Not Applicable | **Weak** |
| Waszak et al. (2018) | Weak | Moderate | Not Applicable | Weak | Strong | Weak | **Weak** |
